# Synergistic immunotoxin-paclitaxel combination against lymphoma

**DOI:** 10.18632/aging.101251

**Published:** 2017-06-13

**Authors:** Fabian Müller

**Affiliations:** Department of Hematology/Oncology, University Hospital Erlangen, Erlangen, Germany

**Keywords:** immunotoxin, paclitaxel, B-NHL, leukemia, targeted therapy, drug synergy, systemic xenograft model

Recombinant immunotoxins are fusion proteins of an antibody and Pseudomonas exotoxin A (PE) [1]. Immunotoxins attack cancer cells specifically while healthy tissue is spared. After internalization, immunotoxins traverse various intracellular compartments to reach the cytosol, where they ADP-ribosylate the eukaryotic elongation factor 2 resulting in an arrest of protein synthesis and the death of target cells. Killing cancer cells by arresting protein synthesis is a unique mechanism among chemotherapeutic drugs which commonly interfere with nucleotide synthesis or microtubule dynamics [1].

*In vitro*, CD22-targeted immunotoxins are cytotoxic in the low pico-molar range against B-cell malignancies including hairy cell leukemia (HCL), chronic lymphocytic leukemia (CLL), acute lymphoblastic leukemia (ALL), and mantle cell lymphoma (MCL) [2]. Additionally, immunotoxins are active in animal models of various B-cell malignancies [1, 2] and the CD22-targeted immunotoxin Moxetumomab pasudotox has been shown to achieve complete remission in half of the patients with therapy-refractory HCL [3]. Despite their substantial activity *in vitro*, CD22-targeted immunotoxins result in fewer than expected responses in patients with ALL [4] and fail to produce responses in CD22-positive non-Hodgkin lymphoma patients [3].

A possible explanation for the low response rate in these patients is the development of neutralizing anti-drug antibodies (ADAs). Immunotoxins, which are derived from a bacterial toxin, are immunogenic in mammals and ADAs form quickly. In patients with hematologic malignancies however, the immune system is compromised by the underlying disease and the development of ADAs takes up to several months [3]. Furthermore, patients who are ADA positive frequently continue to respond [3]. ADAs therefore may not sufficiently explain the lower than expected response rate of CD22-targeted immunotoxins in ALL and lymphoma patients.

Immunotoxins are small proteins which are rapidly cleared from the blood resulting in a short serum half-life of approximately 30 minutes [4]. Because immunotoxins are currently administered as bolus doses, the blood concentration falls quickly below active levels. As recently described, the time that cells have to be exposed to immunotoxin for them to die varies substantially from only a few minutes to more than four days [4, 5]. Cells that respond only if exposed to immunotoxin for more than 5 serum half-times cannot respond *in vivo* because blood levels fall too quickly and the exposure time is too short [4]. By replacing bolus doses with the continuous administration, responses after only one treatment cycle improved greatly from a stable disease after bolus doses to a more than 10-fold reduction of the tumor mass in both, systemic ALL [4] and MCL [5] xenograft models. These data strongly suggest that patients may respond substantially better if treated with continuously administered immunotoxin rather than the currently used bolus doses.

While the maximally tolerated dose of immunotoxin given intravenously results in a high peak serum concentration, the steady state serum concentration of a similar amount given continuously is much lower. The area under the curve (AUC) however is similar possibly explaining the similar off-target toxicity in mice which do not express human CD22 [5]. In patients, the blood counts of healthy B-cells remain unchanged after bolus doses of CD22-targeted immunotoxin [3]. Because the continuous administration increases the on-target activity against malignant B-cells, it is reasonable to also expect an increase of on-target activity against healthy B-cells expressing CD22. Depletion of healthy B-cells results in reduced serum antibody titers [2], which might slow the development of ADAs possibly further increasing the clinical efficacy of CD22-targeted immunotoxins. When the development of ADAs against mesothelin-targeted immunotoxins was suppressed by a co-administration of chemotherapy, the anti-tumor activity was increased and patients showed strong clinical evidence for the induction of an anti-cancer immune reaction [6]. Improved CD22-targeted immunotoxins may similarly induce an anti-lymphoma immune response which should be monitored in future clinical trials.

As a separate approach to improve clinical responses, several groups searched for drugs that synergistically enhance immunotoxins [1, 2]. Paclitaxel was identified as a potent enhancer of bolus doses of mesothelin-targeted immunotoxin *in vivo* resulting in a new clinical trial testing the combination against solid tumors (NCT02810418) [7]. In line with these results, the combination of paclitaxel and CD22-targeted immunotoxin achieved a long-lasting durable remission in 60% of the mice bearing a systemic ALL xenograft and the efficacy of continuously administered immunotoxin was enhanced substantially by 100-fold in a systemic MCL mouse model [5]. These striking effects of the combination of continuously administered immunotoxin and paclitaxel strongly support future clinical trials in patients with B-cell malignancies [5].

Even though numerous novel therapies against B-cell malignancies are rapidly translated into clinical use including chimeric antigen receptor (CAR) T-cells, bispecific T-cell engagers (BiTEs), or small molecules targeting B-cell receptor signaling [2], our recent advances suggest that the well-tolerated CD22-targeted immunotoxins with their unique mode of action may become a future therapeutic option for more than HCL patients.

## References

[1] Pastan I (2006). Nat Rev Cancer.

[2] Wayne AS (2014). Blood.

[3] Kreitman RJ (2012). J Clin Oncol.

[4] Müller F (2016). Clin Cancer Res.

[5] Müller F (2017). Oncotarget.

[6] Hassan R (2013). Sci Transl Med.

[7] Kolyvas E (2017). Oncotarget.

